# Impact of LncRNA GAS5 Genetic Variants and the Epidermal Growth Factor Receptor Phenotypes on the Clinicopathological Characteristics of Lung Adenocarcinoma Patients

**DOI:** 10.3390/ijerph19169971

**Published:** 2022-08-12

**Authors:** Ming-Hong Hsieh, Yi-Liang Wu, Thomas Chang-Yao Tsao, Yi-Wen Huang, Jian-Cheng Lin, Chia-Yi Lee, Ming-Ju Hsieh, Shun-Fa Yang

**Affiliations:** 1 School of Medicine, Chung Shan Medical University, Taichung 402, Taiwan; 2 Department of Psychiatry, Chung Shan Medical University Hospital, Taichung 402, Taiwan; 3 Department of Surgery, Chung Shan Medical University Hospital, Taichung 402, Taiwan; 4 Division of Chest, Department of Internal Medicine, Chung Shan Medical University Hospital, Taichung 402, Taiwan; 5 Department of Health, Pulmonary and Critical Care Unit, Changhua Hospital, Changhua 513, Taiwan; 6 Institute of Medicine, Chung Shan Medical University, Taichung 402, Taiwan; 7 Department of Medical Research, Chung Shan Medical University Hospital, Taichung 402, Taiwan; 8 Nobel Eye Institute, Taipei 115, Taiwan; 9 Oral Cancer Research Center, Changhua Christian Hospital, Changhua 500, Taiwan; 10 Graduate Institute of Biomedical Sciences, China Medical University, Taichung 404, Taiwan; 11 Department of Post-Baccalaureate Medicine, College of Medicine, National Chung Hsing University, Taichung 402, Taiwan

**Keywords:** lung cancer, GAS5, polymorphism, EGFR mutation

## Abstract

The aim of the current study was to evaluate the combined effect of the single nucleotide polymorphism (SNP) in long non-coding RNA growth arrest-specific 5 (GAS5) and the phenotypes of epidermal growth factor receptor (EGFR) on the clinicopathological characteristics of lung adenocarcinoma. The present study examined the relationship between the *GAS5* single-nucleotide polymorphisms (SNPs; rs145204276 Ins/Del, rs55829688 T/C) and the clinicopathological factors in 539 lung adenocarcinoma patients with or without EGFR mutations. We found that the genotype distributions of the two *GAS5* SNPs between different EGFR genotypes were similar after adjusting for age, gender and smoking history. The *GAS5* SNP rs145204276 Ins/Del + Del/Del illustrated a higher distribution with an advanced tumor stage (*p* = 0.030), larger tumor T status (*p* = 0.019), positive lymph node status (*p* = 0.014) and distal metastases (*p* = 0.011) in the EGFR wild type group. In the subgroup analysis of the EGFR wild type population, the presence of *GAS5* SNP rs145204276 Ins/Del + Del/Del was correlated to an advanced tumor stage (*p* = 0.014) and distal metastases (*p* = 0.020) in non-smokers. In conclusion, these data indicate that the *GAS5* SNP rs145204276 variant may help predict tumor stage, lymph node metastasis and distal metastases in patients with EGFR wild type lung adenocarcinoma.

## 1. Introduction

Lung adenocarcinoma is a prevalent neoplasm, worldwide which shows a high incidence in all the lung cancer in the Eastern Asian population compared to the American population [1,2]. The treatments for lung adenocarcinoma today include surgical excision, chemotherapy and target therapy [3,4]. Regarding the risk factors for the lung adenocarcinoma, cigarette smoking is a well-known risk factor for lung cancer including adenocarcinoma in which the presence of cigarette smoking history can elevate the risk of lung cancer development about 20–50-fold [5]. Besides, a recent article demonstrated that the male gender produces a significantly higher risk of grade three lung adenocarcinoma compared to the female population [6].

Certain genetic factors are associated with the development of lung adenocarcinoma and the epidermal growth factor receptor (EGFR) and its mutation is probably the genetic risk factor of lung adenocarcinoma that has been studied most completely [7]. The EGFR mutation L858R expression and exon 19 in-frame deletion are correlated to a higher sensitivity of lung cancer to EGFR tyrosine kinase inhibitors [8]. In addition, the presence of EGFR mutations and the single nucleotide polymorphism (SNP) of Aurora kinase A is associated with earlier tumor stage of the lung adenocarcinoma [9]. Except the EGFR, the long non-coding RNA H19 causes the progression and metastasis of lung cancer [10], and the LAMC1 SNP rs3768617 could also increase the risk of lung cancer [11].

The growth arrest-specific 5 (GAS5) is a type of long non-coding RNA that acts as a tumor suppressor and influences the cell apoptosis and tumorigenesis processes in accordance with previous publications [12,13,14]. Regarding the SNP of *GAS5*, the *GAS5* SNP rs145204276 increases the probability of larger tumor status in urothelial cell carcinoma [13]. In addition, the *GAS5* SNP rs145204276 is significantly correlated with lung cancer susceptibility [15]. However, there are scant studies that discuss the SNP of *GAS5* and its correlation to the clinical features of lung adenocarcinoma. Also, because the SNPs of other gene would interact with EGFR genotype and affect the characters of lung adenocarcinoma [9], a similar action may occur between *GAS5* SNPs and EGFR which need additional evaluation.

Consequently, the purpose of the current study was to evaluate the effect of *GAS5* SNP plus EGFR phenotypes on the clinical manifestations of lung adenocarcinoma. In addition, the subgroup analysis according to the cigarette smoking history in EGFR wild type population was conducted.

## 2. Materials and Methods

### 2.1. Patients and Specimens

This research enrolled patients with lung adenocarcinoma and followed up for more than six months in Chung Shan Medical University Hospital and Changhua Hospital and a total of 539 patients were included in the study population. These patients were further categorized into those with EGFR wild type and EGFR mutation, and 221 and 318 patients constituted the two groups respectively. The EGFR mutation in this study included the L858R expression and exon 19 in-frame deletion. The age, gender, and cigarette smoking history of these patients was obtained and a cancer sample of all participants was collected as the form of frozen specimen. Then Tumor, Node, Metastasis (TNM) condition, tumor stages, degree of cell differentiation (i.e., the well-differentiated, the moderately differentiated, and the poorly differentiated) were based on the American Joint Committee on Cancer. This study was adhered to the Declaration of Helsinki in 1964 and later amendments and Institutional Review Boards of Chung Shan Medical University Hospital approved this study (Project code: No. CS18173; 18 February 2019 and CS1-20144, 24 August 2020). Besides, written informed consents were taken from all the participants after explaining and discussing the details of this research. For *GAS5* SNP analyses, we took venous blood and preserved it in ethylenediaminetetraacetic acid-containing tubes. The samples were centrifuged and restored in one laboratory refrigerator at −80 degrees Celsius.

### 2.2. Genomic DNA Extraction and Analysis of GAS5 SNP via Real-Time PCR

Two SNPs of *GAS5*: rs145204276 (Ins/Del) and rs55829688 (T/C) were chosen due to their effects on lung cancer and other neoplasms [15,16,17]. The genotyping procedure is referred to in our previous research [18]. The genomic DNA was extracted from leukocytes in venous blood sample via QIAamp DNA kits (Qiagen, Valencia, CA, USA), and all steps with QIAamp DNA kits were in accordance with manufacture’s instruction. The reason we used leukocytes to take genomic DNA is because it is a simple way to obtain human genomic DNA. Isolated DNA was put in one refrigerator under −20 degrees Celsius. The two *GAS5* SNPs were examined via the ABI StepOne Real-Time PCR System (Applied Biosystems, Foster City, CA, USA). After that, the polymorphisms of both *GAS5* SNPs were analyzed with the assistance of the TaqMan assay technique as well as the SDS version 3.0 software (Applied Biosystems) to enhance Real-Time PCR completeness.

### 2.3. Statistical Analysis

The SAS version 9.4 (SAS Institute Inc., Cary, NC, USA) was applied. Descriptive analyses such as mean value, standard deviation (SD) and percentage were used to reveal basic characters and laboratory data of the study population. The Chi-squared test and independent *t*-test were applied for comparing each value between EGFR wild type and EGFR mutation groups. The adjusted odds ratio (AOR) with a 95% confidence interval (CI) of genotype distribution between the EGFR wild type and EGFR mutation population was produced via multiple logistic regression models after controlling for age, gender and cigarette smoking status. In further analyses, the genotypes of rs145204276 (Ins/Del) and rs55829688 (T/C) and their effects on clinicopathological characteristics of lung adenocarcinoma in the EGFR wild type and EGFR mutation population were calculated with corresponding odds ratio (OR) and 95% CI. For the subgroup analysis, the EGFR wild type patients were categorized as non-smokers or ever-smokers and the relationship between *GAS5* rs145204276 genotype frequencies and clinicopathological characteristics of lung adenocarcinoma were estimated. The statistically significant level was defined as *p* < 0.05.

## 3. Results

### 3.1. Basic Characteristics between EGFR Wild Type and EGFR Mutation Groups

The mean age in the EGFR wild type group was 64.63 ± 12.18 years old which was similar to the mean age in the EGFR mutation group (65.45 ± 12.55) (*p* = 0.453). On the other hand, a higher female ratio (66.7 versus 38.5%, *p* = 0.001) and lower cigarette smoking rate (19.5 versus 52.9%, *p* = 0.001) were found in the EGFR mutation group. Except for a lower percentage of poor cell differentiation in the EGFR mutation group (*p* < 0.001), all tumor characteristics were identical between the two groups (Table 1).

### 3.2. Distribution of GAS5 SNPs and EGFR Genotypes in Different Clinical Characters of Lung Adenocarcinoma

The distribution frequencies of *GAS5* SNP rs145204276 (Ins/Del) and rs55829688 (T/C) between the EGFR wild type and EGFR mutation groups are shown in Table 2. After adjusting for age, gender and cigarette smoking history, the genotype distributions of the two *GAS5* SNPs between different EGFR genotypes were similar (all *p* > 0.05). About the genetic polymorphisms and the clinicopathologic characteristics of lung adenocarcinoma, the *GAS5* SNP rs145204276 Ins/Del + Del/Del demonstrated a higher distribution in advanced tumor stage (*p* = 0.030), larger tumor T status (*p* = 0.019), positive lymph node status (*p* = 0.014) and the presence of distal metastasis (*p* = 0.011) when compared to the *GAS5* rs145204276 wild type in the EGFR wild type group (Table 3). On the other hand, there may not be strong relationships between the distribution frequencies of *GAS5* SNP rs55829688 and the clinicopathologic characteristics of lung adenocarcinoma in all EGFR genotypes (all *p* > 0.05) (Table 4).

The AORs with 95% CIs were estimated by multiple logistic regression models after controlling for age, gender and cigarette smoking status.

### 3.3. Subgroup Analysis about the Cigarette Smoking Status and GAS5 SNP rs145204276 in EGFR Wild Type

In the subgroup analyses in the EGFR wild type population, the presence of *GAS5* SNP rs145204276 Ins/Del + Del/Del may be correlated with an advanced tumor stage (*p* = 0.014) and distal metastases (*p* = 0.020) in non-smoker individuals. Moreover, a higher ratio of GAS5 SNP rs145204276 Ins/Del + Del/Del variant was found in the patients with lymph node invasion and cigarette smoking history (*p* = 0.036). The rest of the outcomes of the subgroup analyses concerning the cigarette smoking status revealed insignificant values (all *p* > 0.05) (Table 5).

## 4. Discussion

In our study, the *GAS5* SNP rs145204276 Ins/Del + Del/Del contributed to worse tumor condition concerning the tumor stage, tumor T status and lymph node status in lung adenocarcinoma patients with EGFR wild type. In more details, the *GAS5* SNP rs145204276 Ins/Del + Del/Del causes a higher tumor stage and leads to distal metastases in non-smokers with lung adenocarcinoma and wild-type EGFR.

A number of studies had evaluated the association between EGFR and lung adenocarcinoma previously [7]. The two common EGFR mutations that influence the clinical characteristics of lung adenocarcinoma are the L858R expression and exon 19 in-frame deletion [19,20]. According to previous literature, the existence of EGFR L858R expression mutation may cause a better therapeutic outcome of lung adenocarcinoma without other genetic risk factors such as cytoplasmic ERβ1 expression [21]. However, intensive EGFR L858R expression on micropapillary components may increase the recurrence of lung adenocarcinoma [22]. The exon 19 in-frame deletion is another important genetic variant for the clinical course of lung adenocarcinoma in which the EGFR exon 19 in-frame deletion produced a better response to gefitinib and erlotinib in patients with lung adenocarcinoma [23], and another study illustrated that the overall survival in advanced lung adenocarcinoma patients with EGFR exon 19 in-frame deletions was better than those with EGFR L858R expression [24]. In addition, the EGFR exon 20 insertion mutations may change the resistance of lung cancer to EGFR inhibitors [25]. In addition to the genetic variant itself, the EGFR genotypes could show different effects on the clinicopathologic characters of lung adenocarcinoma when combining it with other genetic polymorphisms. For instance, the Aurora kinase A SNP rs6024836 AG + GG is related to an earlier tumor stage if combined with the presence of an EGFR mutation, L858R expression and exon 19 in-frame deletion [9]. Besides, the LncRNA H19 SNP rs217727 are related to advanced tumor status for lung adenocarcinoma with EGFR wild type [26]. On the other side, the GAS5 and its SNP is another genetic risk factor for cardiovascular disease and various malignancies including the lung adenocarcinoma [13,15,27,28,29]. In the study by Liang et al., the GAS5 expression would reduce in non-small cell lung cancer, which can serve as a biomarker [30], and the GAS5 would lead to lower rate of lung adenocarcinoma invasion and migration [31]. Consequently, the genotypes of these two genetic factors, the EGFR and *GAS5*, may interact and alter the clinical course of lung adenocarcinoma. The above hypothesis is supported by the results of this study at least to some extent.

Concerning the genetic polymorphism of *GAS5* and the clinical characters of lung adenocarcinoma with different EGFR genotypes, the lung adenocarcinoma patients with EGFR wild type and *GAS5* SNP rs145204276 Ins/Del + Del/Del would experience higher tumor stage, larger tumor T status, advanced lymph node invasion and distal metastasis compared to those patients without the *GAS5* variant. This is a relatively new finding that the SNP of *GAS5* can grossly affect the clinicopathological characteristics of lung adenocarcinoma with wild-type EGFR. In other type of cancer, although the *GAS5* SNP rs145204276 is related to a lower incidence of tumor progression in gastric cancer and serves as a protector [16,32], the existence of *GAS5* SNP rs145204276 may contribute to a worse tumor stage and size in certain subjects with oral cancer [17]. In addition, the GAS5 is a potential target for breast cancer treatment [33]. For the aspect of EGFR, the EGFR wild type is associated with poorer treatment response and progression-free survival compared to their mutation counterpart [34]. Consequently, the double tumorigenic effects of the two genes could increase the tumor progression in the lung adenocarcinoma. On the contrary, the combined effect of EGFR wild type and *GAS5* SNP rs145204276 Ins/Del + Del/Del increases neither the percentage of poorly differentiated tumor cells, nor the cell differentiation of the EGFR mutation type. Moreover, the *GAS5* SNP rs55829688 TC+CC did not alter all the clinical manifestations of lung adenocarcinoma with all types of the EGFR genotype. The above two findings may indicate that the influence of GAS5 on lung adenocarcinoma is not universal.

In the subgroup analyses of EGFR wild type, the *GAS5* SNP rs145204276 Ins/Del + Del/Del are related the tumor stage and distal metastases of lung adenocarcinoma in the non-smoker population and enhance the lymph node invasion of lung adenocarcinoma in the ever-smoker group. Seldom research has reported this specific effect of *GAS5* SNP rs145204276 Ins/Del + Del/Del in patients with different smoking histories. The exact mechanism for this difference remains unknown; maybe the tobacco particles would alter the lymph node condition and make it more vulnerable for lung adenocarcinoma cells under certain genetic polymorphisms. Additionally, the SNPs at 22q12 and the 15q15.2 locus would increase the risk of lung cancer even in non-smokers [35]; maybe the *GAS5* SNP rs145204276 Ins/Del + Del/Del owns a similar effect to them. On the other side, all the ratios of worse tumor statuses including tumor stage, tumor T status, lymph node status and distal metastases were silently higher with the presence of *GAS5* SNP rs145204276 Ins/Del + Del/Del compared to the *GAS5* SNP rs145204276 Ins/Ins wild type. This finding may further imply the prominent effect of *GAS5* SNP rs145204276 on the TNM score and tumor stage of lung adenocarcinoma which is in accordance with our findings in the above paragraph. Still, whether the length of cigarette smoking period would interact with *GAS5* SNP rs145204276 and alter the clinicopathological characteristics of lung adenocarcinoma needs further validation.

There are certain limitations existing in this study. Firstly, the cross-sectional nature of the study design makes the longitudinal follow up to survey the treatment outcome in different groups and genotypes impossible, and further cohort research that enrolls participants with different genotypes should be conducted. Secondly, the number of patients (539) enrolled in the current study is relatively small compared to the population-based studies that often recruit more than 1000 patients. This smaller sample size may contribute to some bias despite the power of our study population having reached 0.97 and no recruitment difficulty being encountered. Besides, the air-pollution particle is an important risk factor for the lung adenocarcinoma development [1]; we did not collect this data and put it into the multivariable analyses, which may influence the statistical outcome in this study. Nevertheless, all the patients included in this study were from Taichung and Chunghua city in Taiwan, which is an area with severe air pollution. As a consequence, the impact of air-pollution particle exposure on the outcome evaluation in this study might be minimal.

## 5. Conclusions

In conclusion, the presence of the *GAS5* SNP rs145204276 variant could lead to advanced clinicopathological characteristics of lung adenocarcinoma under the presence of the EGFR wild type. Furthermore, the affected clinicopathological characters by *GAS5* SNP rs145204276 are different between the non-smokers and ever-smokers. Consequently, the *GAS5* genotype analyses in patients with lung adenocarcinoma and EGFR wild type may be suggested to find advanced tumor condition. Further large-scale prospective studies to evaluate whether the *GAS5* SNP rs145204276 and other variants would affect the therapeutic outcome in lung adenocarcinoma with wild-type wild type are mandatory.

## Figures and Tables

**Table 1:** Demographics and clinical characteristics of 539 patients in lung adenocarcinoma with EGFR mutation status.

Variable	EGFR Wild Type (N = 221) *n* (%)	EGFR Mutation (N = 318) *n* (%)	*p* Value
Age			
Mean ± SD	64.63 ± 12.18	65.45 ± 12.55	0.453
Gender			
Male	136 (61.5%)	106 (33.3%)	0.001 *
Female	85 (38.5%)	212 (66.7%)	
Cigarette smoking status			
Never-smoker	104 (47.1%)	256 (80.5%)	0.001 *
Ever-smoker	117 (52.9%)	62 (19.5%)	
Stage			
I+II	49 (22.2%)	76 (23.9%)	0.640
III+IV	172 (77.8%)	242 (76.1%)	
Tumor T status			
T1+T2	109 (49.3%)	171 (53.8%)	0.309
T3+T4	112 (50.7%)	147 (46.2%)	
Lymph node status			
Negative	60 (27.1%)	89 (28.0%)	0.831
Positive	161 (72.9%)	229 (72.0%)	
Distant metastasis			
Negative	98 (44.3%)	127 (39.9%)	0.308
Positive	123 (55.7%)	191 (60.1%)	
Cell differentiation			
Well	19 (8.6%)	35 (11.0%)	<0.001 *
Moderately	136 (61.5%)	248 (78.0%)	
Poorly	66 (29.9%)	35 (11.0%)	

N: number; SD: standard deviation; * Denotes significant difference between the two groups.

**Table 2:** Distribution frequency of *GAS5* genotypes of patients with lung adenocarcinoma and multiple logistic regression analysis of EGFR mutation association.

Genotypes	EGFR Wild Type (N = 221)	EGFR Mutation (N = 318)	AOR (95% CI)	*p* Value
rs145204276				
Ins/Ins	92 (41.6%)	131 (41.2%)	1.00	
Ins/Del	102 (46.2%)	144 (45.3%)	0.973 (0.655–1.445)	0.891
Del/Del	27 (12.2%)	43 (13.5%)	1.064 (0.590–1.920)	0.836
Ins/Del + Del/Del	129 (58.4%)	187 (58.8%)	0.992 (0.682–1.443)	0.967
rs55829688				
TT	100 (45.2%)	166 (52.2%)	1.00	
TC	99 (44.8%)	121 (38.1%)	0.787 (0.532–1.163)	0.229
CC	22 (10.0%)	31 (9.7%)	0.678 (0.356–1.293)	0.238
TC + CC	121 (54.8%)	152 (47.8%)	0.765 (0.528–1.108)	0.157

N: number; AOR: adjusted odds ratio; CI: confidence interval.

**Table 3:** Clinicopathologic characteristics of lung adenocarcinoma patients with EGFR mutations, stratified by polymorphic genotypes of GAS5 rs145204276.

Variable	ALL (N = 539)			EGFR Wild type (N = 221)			EGFR Mutation (N = 318)		
	Ins/Ins (N = 223)	Ins/Del + Del/Del (N = 316)	*p* Value	Ins/Ins (N = 92)	Ins/Del + Del/Del (N = 129)	*p* Value	Ins/Ins (N = 131)	Ins/Del + Del/Del (N = 187)	*p* Value
Stages									
I+II	53 (23.8%)	72 (22.8%)	0.790	27 (29.3%)	22 (17.1%)	0.030 *	26 (19.8%)	50 (26.7%)	0.156
III+IV	170 (76.2%)	244 (77.2%)		65 (70.7%)	107 (82.9%)		105 (80.2%)	137 (73.3%)	
Tumor T status									
T1+T2	126 (56.5%)	154 (48.7%)	0.075	54 (58.7%)	55 (42.6%)	0.019 *	72 (55.0%)	99 (52.9%)	0.722
T3+T4	97 (43.5%)	162 (51.3%)		38 (41.3%)	74 (57.4%)		59 (45.0%)	88 (47.1%)	
Lymph node status									
Negative	65 (29.1%)	84 (26.6%)	0.512	33 (35.9%)	27 (20.9%)	0.014 *	32 (24.4%)	57 (30.5%)	0.237
Positive	158 (70.9%)	232 (73.4%)		59 (64.1%)	102 (79.1%)		99 (75.6%)	130 (69.5%)	
Distant metastasis									
Negative	99 (44.4%)	126 (39.9%)	0.294	50 (54.3%)	48 (37.2%)	0.011 *	49 (37.4%)	78 (41.7%)	0.440
Positive	124 (55.6%)	190 (60.1%)		42 (45.7%)	81 (62.8%)		82 (62.6%)	109 (58.3%)	
Cell differentiation									
Well/Moderately	179 (80.3%)	259 (82.0%)	0.620	62 (67.4%)	93 (72.1%)	0.452	117 (89.3%)	166 (88.8%)	0.879
Poorly	44 (19.7%)	57 (18.0%)		30 (32.6%)	36 (27.9%)		14 (10.7%)	21 (11.2%)	

N: number; * Denotes significant difference between the two genotypes.

**Table 4:** Clinicopathologic characteristics of lung adenocarcinoma patients with EGFR mutations, stratified by polymorphic genotypes of GAS5 rs55829688.

Variable	ALL (N = 539)			EGFR Wild Type (N = 221)			EGFR Mutation (N = 318)		
	TT (N = 266)	TC+CC (N = 273)	*p* Value	TT (N = 100)	TC+CC (N = 121)	*p* Value	TT (N = 166)	TC+CC (N = 152)	*p* Value
Stages									
I+II	70 (26.3%)	55 (20.1%)	0.090	25 (25.0%)	24 (19.8%)	0.358	45 (27.1%)	31 (20.4%)	0.161
III+IV	196 (73.7%)	218 (79.9%)		75 (75.0%)	97 (80.2%)		121 (72.9%)	121 (79.6%)	
Tumor T status									
T1+T2	144 (54.1%)	136 (49.8%)	0.316	52 (52.0%)	57 (47.1%)	0.469	92 (55.4%)	79 (52.0%)	0.538
T3+T4	122 (45.9%)	137 (50.2%)		48 (48.0%)	64 (52.9%)		74 (44.6%)	73 (48.0%)	
Lymph node status									
Negative	80 (30.1%)	69 (25.3%)	0.213	28 (28.0%)	32 (26.4%)	0.796	52 (31.3%)	37 (24.3%)	0.166
Positive	186 (69.9%)	204 (74.7%)		72 (72.0%)	89 (73.6%)		114 (68.7%)	115 (75.7%)	
Distant metastasis									
Negative	119 (44.7%)	106 (38.8%)	0.164	50 (50.0%)	48 (39.7%)	0.124	69 (41.6%)	58 (38.2%)	0.535
Positive	147 (55.3%)	167 (61.2%)		50 (50.0%)	73 (60.3%)		97 (58.4%)	94 (61.8%)	
Cell differentiation									
Well/Moderately	217 (81.6%)	221 (81.0%)	0.852	74 (74.0%)	81 (66.9%)	0.254	143 (86.1%)	140 (92.1%)	0.090
Poorly	49 (18.4%)	52 (19.0%)		21 (26.0%)	47 (33.1%)		23 (13.9%)	12 (7.9%)	

N: number.

**Table 5:** GAS5 rs145204276 genotype distribution and clinicopathologic characteristics of EGFR wild type lung adenocarcinoma patients with different cigarette smoking statuses.

Variable	Non-Smoker (N = 104)			Ever-Smoker (N = 117)		
	Ins/Ins (N = 39)	Ins/Del + Del/Del (N = 65)	*p* Value	Ins/Ins (N = 53)	Ins/Del + Del/Del (N = 64)	*p* Value
Stages						
I+II	15 (38.5%)	11 (16.9%)	0.014 *	12 (22.6%)	11 (17.2%)	0.460
III+IV	24 (61.5%)	54 (83.1%)		41 (77.4%)	53 (82.8%)	
Tumor T status						
T1+T2	23 (59.0%)	27 (41.5%)	0.085	31 (58.5%)	28 (43.7%)	0.112
T3+T4	16 (41.0%)	38 (58.5%)		22 (41.5%)	36 (56.3%)	
Lymph node status						
Negative	15 (38.5%)	16 (24.6%)	0.135	18 (34.0%)	11 (17.2%)	0.036 *
Positive	24 (61.5%)	49 (75.4%)		35 (66.0%)	53 (82.8%)	
Distant metastasis						
Negative	21 (53.8%)	20 (30.8%)	0.020 *	29 (54.7%)	28 (43.7%)	0.237
Positive	18 (46.2%)	45 (69.2%)		24 (45.3%)	36 (56.3%)	
Cell differentiation						
Well/Moderately	27 (69.2%)	48 (73.8%)	0.611	35 (66.0%)	45 (70.3%)	0.621
Poorly	12 (30.8%)	17 (26.2%)		18 (34.0%)	19 (29.7%)	

N: number; * Denotes significant difference between the two genotypes.

## Data Availability

The datasets generated for this study are available on request to the corresponding authors.
